# Heavy metals in the Galápagos Archipelago: a One Health scoping review

**DOI:** 10.3389/fpubh.2026.1896419

**Published:** 2026-10-28

**Authors:** Jesús Endara-Mina, Tony Fernando Cepeda-Reza, Alexandra Mishell Villagómez-López, Cristopher-Josué Escudero, Dario Chicaiza, Jhon Paul Guerrero-González, Pedro Isaac Torres-Balarezo, Carlos Astudillo-Abad, Ricardo Flores-Palomino, Bryan Moposita, Andrea Salgado, Sebastián Fuentes-Villarreal, Stefan Alexander Brück

**Affiliations:** 1 Facultad de Ciencias de la Salud, Universidad Técnica Particular de Loja (UTPL), Loja, Ecuador; 2 Hospital Provincial General Pablo Arturo Suárez, Ministerio de Salud Pública, Quito, Ecuador; 3 Facultad de Ciencias Médicas, Universidad Central del Ecuador (UCE), Quito, Ecuador; 4 Hospital General del Sur de Quito, Instituto Ecuatoriano de Seguridad Social, Quito, Ecuador; 5 Hospital Básico de Paute, Ministerio de Salud Pública del Ecuador, Paute, Ecuador; 6 Facultad de Ciencias Médicas de la Salud y de la Vida, Universidad Internacional del Ecuador, Quito, Ecuador; 7 Facultad de Ciencias de la Salud y Bienestar Humano, Universidad Tecnológica Indoamérica, Quito, Ecuador; 8 Facultad de Ciencias Biológicas, Universidad Central del Ecuador, Quito, Ecuador

**Keywords:** bioaccumulation, biomagnification, ecotoxicology, Galápagos Marine Reserve, heavy metals, One Health, wildlife biomonitoring

## Abstract

**Background:**

Heavy metal contamination is a global concern due to its persistence, bioaccumulation, and toxicity to ecosystems and human health. The Galápagos Archipelago, with its high endemism and limited industrial activity, provides a unique setting where natural and anthropogenic sources may interact. This review aimed to synthesize evidence on the presence, distribution, sources, and effects of heavy metals in environmental matrices and wildlife in the Galápagos.

**Methods:**

A systematic search was conducted in PubMed, Scopus, and Web of Science. Studies evaluating heavy metals and metalloids in environmental matrices and terrestrial or marine organisms within the archipelago were included, without restrictions on language or publication year.

**Results:**

Evidence was mainly concentrated in environmental compartments (soils, sediments, water, and fumarolic systems) and wildlife, particularly fish, seabirds, pinnipeds, and invertebrates. The most frequently assessed metals were mercury, cadmium, lead and copper. Cadmium exceeded EFSA maximum levels in all evaluated commercial fish species, posing the highest risk in *Caulolatilus affinis*. Mercury exceeded EFSA/FDA/Codex limits in *Acanthocybium solandri, Pontinus clemensi*, and *Hyporthodus mystacinus*. Furthermore, mercury in *Grapsus grapsus* from San Cristóbal exceeded the EPA threshold, and was detected in feathers of 8 seabird species, showing higher levels during La Niña upwelling. Lead in *Zalophus wollebaeki* bone was lower than in Mauritania and Uruguay, which act as relatively unpolluted references. Both natural and localized anthropogenic sources were identified, including agriculture, phosphate fertilizers, and maritime activities.

**Conclusions:**

Important gaps persist, particularly in human biomonitoring and integrated surveillance. Future research should adopt a One Health approach to better link environmental contamination with ecological and public health outcomes.

## 1 Introduction

Heavy metal contamination represents a growing global environmental issue that compromises ecosystem integrity and disrupts fundamental biological processes (1–4). Metals are characterized as good conductors of heat and electricity (5, 6), possess high density, and, at room temperature, generally occur in a solid state, with the exception of mercury and gallium (7). Furthermore, their salts have the capacity to form electropositive ions (cations) in solution; in the environment, metals may occur as pure elements or as components of widely used alloys such as steel and bronze (8, 9).

Within this group, the term heavy metals (HMs) is operationally used to describe a heterogeneous set of metallic elements and metalloids characterized by high density, environmental persistence, and potential environmental toxicity, rather than by a single physical criterion (10). Although heavy metals have traditionally been defined as elements with a density at least five times greater than that of water, generally ranging between approximately 4 and 7 g/cm^3^ (11), this classification is limited and has been increasingly questioned in the scientific literature. In the context of environmental contamination and health, primary interest lies in their toxicological behavior, persistence, and capacity for bioaccumulation and biomagnification, regardless of their exact density (12–15).

Potentially toxic elements can occur in the environment in different chemical forms, which determine their bioavailability, environmental behavior, and toxicity. Among the most relevant in insular ecosystems are mercury (Hg), arsenic (As), cadmium (Cd), lead (Pb), copper (Cu), and zinc (Zn). Mercury, particularly in the form of methylmercury (MeHg), is of major concern because of its long-range transport, microbial methylation in aquatic environments, and biomagnification through marine food webs, thereby increasing human exposure through seafood consumption and contributing to neurological, cardiovascular, and reproductive effects (16–18). Given the methodological complexity and higher analytical cost of directly quantifying methylmercury (MeHg), total mercury (THg) is often used as a proxy for MeHg in animal tissues, particularly in fish muscle, where MeHg commonly represents the predominant fraction of accumulated mercury (19).

The trophic transfer of As, Cd, Pb, Cu, and Zn is more variable and depends on chemical speciation, the organism involved, and food-web structure. Although Cu and Zn are essential micronutrients, they may reach toxic concentrations in environments influenced by geogenic, volcanic, or anthropogenic inputs (1, 20).

Understanding the impact of heavy metals requires an appropriate geographical and ecological context. The Galápagos Archipelago is located in the Pacific Ocean, approximately 972 km off the mainland coast of Ecuador, and originated more than four million years ago as a result of active volcanic processes. It comprises 13 large islands (four of which are inhabited) with an area greater than 10 km^2^, six medium-sized islands ranging between 1 and 10 km^2^, and more than 200 small islands and rocky out-crops of varying dimensions (21). This geographic isolation has fostered the development of unique terrestrial and marine ecosystems, regulated by complex ocean–atmosphere interactions characteristic of the eastern equatorial Pacific (22).

From a biological perspective, the Galápagos hosts extraordinary biodiversity. Approximately 2,900 marine species have been described, around 18% of which are endemic; these include 45 species of birds, 42 species of reptiles, six species of mammals, and 79 species of fish found exclusively in the archipelago. The vascular flora comprises approximately 560 species, of which about 180 (32%) are endemic, according to data from the Galápagos National Park (23). At the same time, the archipelago is home to approximately 28,583 inhabitants, according to the 2024 census (24). In recognition of its global ecological significance, Galápagos was designated a Natural World Heritage Site in 1976, a UNESCO Biosphere Reserve in 1984, and a Ramsar Site in 2001 (23).

A complex pattern of heavy metal contamination is known to exist in volcanic island ecosystems, where natural and anthropogenic sources converge. In volcanic areas, elements such as Ni, Cr, Cu, and Zn exhibit naturally elevated concentrations derived from volcanic parent material, while Pb, Cd, Hg, and As show greater influence from human activities, including tourism, transportation, agriculture, and industrial emissions (25–27). Human activities also increase the concentrations of those elements of volcanic origin, particularly in soils near airports and areas of high tourist activity (28, 29). In insular marine ecosystems, sediments and bivalve organisms accumulate heavy metals that biomagnify through food webs, with Cd showing the highest bioaccumulation factors (30, 31). Risk assessments in island populations identify unacceptable carcinogenic risks from exposure to Cr, Ni, and As, especially in children, and elevated risks from consumption of marine products contaminated with As and Cd (30, 32).

The scientific literature has proposed multiple approaches to explain the origin and distribution of heavy metals in the Galápagos ecosystem. Some studies emphasize the contribution of local sources associated with human activities on the islands; others highlight the role of contamination transported by long-distance ocean currents or of external origin; while a substantial body of research underscores the volcanic origin of the archipelago as a significant natural source of metals (33, 34). In this context, integrating both natural and anthropogenic sources and systematizing the available evidence is essential.

Therefore, the objective of the present study is to systematically describe the available evidence regarding the presence, distribution, sources, and effects of heavy metals and their chemical forms in the Galápagos Archipelago, as well as to analyze their potential implications for human health while identifying critical evidence gaps, particularly the absence of human biomonitoring studies, from a One Health perspective.

## 2 Materials and methods

### 2.1 Study design

The present work constitutes a scoping review conducted under a One Health framework, recognizing that exposure to heavy metals in the Galápagos Islands represents a complex socioecological phenomenon integrating risks and consequences for human health, wildlife, marine organisms, and insular ecosystems. This review was carried out following the Joanna Briggs Institute methodology for scoping reviews (35) and was reported in accordance with the Preferred Reporting Items for Systematic Reviews and Meta-Analyses extension for Scoping Reviews (PRISMA-ScR) (36) (Supplementary Table S1).

### 2.2 Eligibility criteria

Eligibility criteria were established following the PCC framework (Population, Concept, Context) (37). Regarding the population, studies including humans, terrestrial or marine fauna (including native and endemic species), as well as environmental matrices and ecosystems of the Galápagos Archipelago potentially exposed to heavy metals were considered. The concept encompassed all research assessing the presence, concentration, distribution, sources, mobility, bioaccumulation, biomagnification, or toxicological effects of heavy metals (such as Hg, MeHg, As, Cd, Pb, Cu, Zn, or other metalloids), whether in human populations, animals, or environmental components, within an integrated One Health perspective. The context was strictly limited to the Galápagos Archipelago, including terrestrial, marine, and coastal environments, protected areas, high-endemism zones, human settlements, and maritime transit areas.

Studies conducted in any island or marine area of the archipelago were eligible, regardless of publication year or language. Studies not conducted in Galápagos, those not assessing heavy metals as the primary exposure, or those lacking primary data or a verifiable methodology were excluded.

### 2.3 Information sources and search strategy

The literature search was conducted systematically using three high-visibility international databases: PubMed, Scopus, and Web of Science. No restrictions were applied regarding language or year of publication, given the exploratory nature of this scoping review and the limited availability of studies on heavy metal contamination in the region.

The search strategy employed combinations of free-text terms related to the heavy metals of interest (e.g., Hg, MeHg, As, Cd, Pb, Cu, Zn), associated environmental and toxicological processes (such as environmental contamination, pollution, bioaccumulation, biomagnification, ecotoxicity), and the specific geographical context of the archipelago (including Galapagos, Galápagos Islands, and Islas Galápagos). These combinations were adapted for each database to maximize search sensitivity and specificity. The full version of each strategy, along with the exact terms used, is provided in Supplementary Table S2.

### 2.4 Study selection and data extraction

Articles were independently assessed by two reviewers using the Rayyan software (38). Duplicate records were identified and removed prior to the screening process. Discrepancies between reviewers were resolved through consensus, and when necessary, a third reviewer was consulted.

A data extraction form was developed and pilot-tested to ensure clarity and consistency. This instrument collected detailed information on the study's geographic location within the Galápagos Archipelago, the environmental or biological matrix analyzed (water, sediments, soil, air, flora, fauna, or human samples), the type of heavy metal assessed and its reported concentrations, the analytical techniques employed (such as ICP–MS, AAS, XRD, spectrophotometry, or remote sensing methods), and the potential natural or anthropogenic sources of contamination identified by the authors.

Additionally, data were extracted on the characteristics of the human, animal, or ecosystem populations studied, including evidence of bioaccumulation, biomagnification, toxicity, or ecological impacts, as well as measured outcomes and the methodological design of each study.

Generative AI (ChatGPT Deep Search, GPT-5.5) was used as a supporting tool during data extraction. All AI-assisted extracted data were subsequently and independently verified against the original full-text articles by two human reviewers to ensure accuracy and consistency. Any discrepancies identified during verification were resolved by consensus before inclusion in the final dataset.

### 2.5 Synthesis of results

Results were synthesized using a structured narrative approach, complemented by descriptive and spatial analysis, consistent with the methodological and thematic heterogeneity of the included studies. Given that the articles encompassed multiple biological and environmental matrices, as well as different metals and analytical designs, findings were organized hierarchically into main domains: (a) marine and wildlife fauna, (b) terrestrial and volcanic environmental matrices, and (c) integrative multidomain evidence under a One Health framework.

Within each domain, results were stratified by matrix type (fish, birds, marine mammals, reptiles, crustaceans, agricultural soils, volcanic soils, hydrothermal systems) and by the metal assessed, prioritizing those most frequently reported (Cd, Hg, Pb, Cu and Zn). Patterns of bioaccumulation, biomagnification, and variability between demersal and pelagic species were described where applicable.

To complement the narrative synthesis, a spatial visualization was developed using the geographic coordinates of the sampling locations reported in the included studies. Spatial interpolation was performed in Python v3.X using a kriging-like approach implemented through Gaussian process regression, and geographic visualization was generated with Matplotlib. Importantly, these interpolated surfaces represent the relative spatial distribution of research effort based on the locations reported in the included studies, rather than measured heavy metal concentrations or contamination intensity. Therefore, the maps should be interpreted as an evidence-mapping tool illustrating areas with greater or lower research coverage across the Galápagos Archipelago. The quantitative synthesis presented in Figure 1 was conducted in R (2026.06.0).

**Figure 1:**
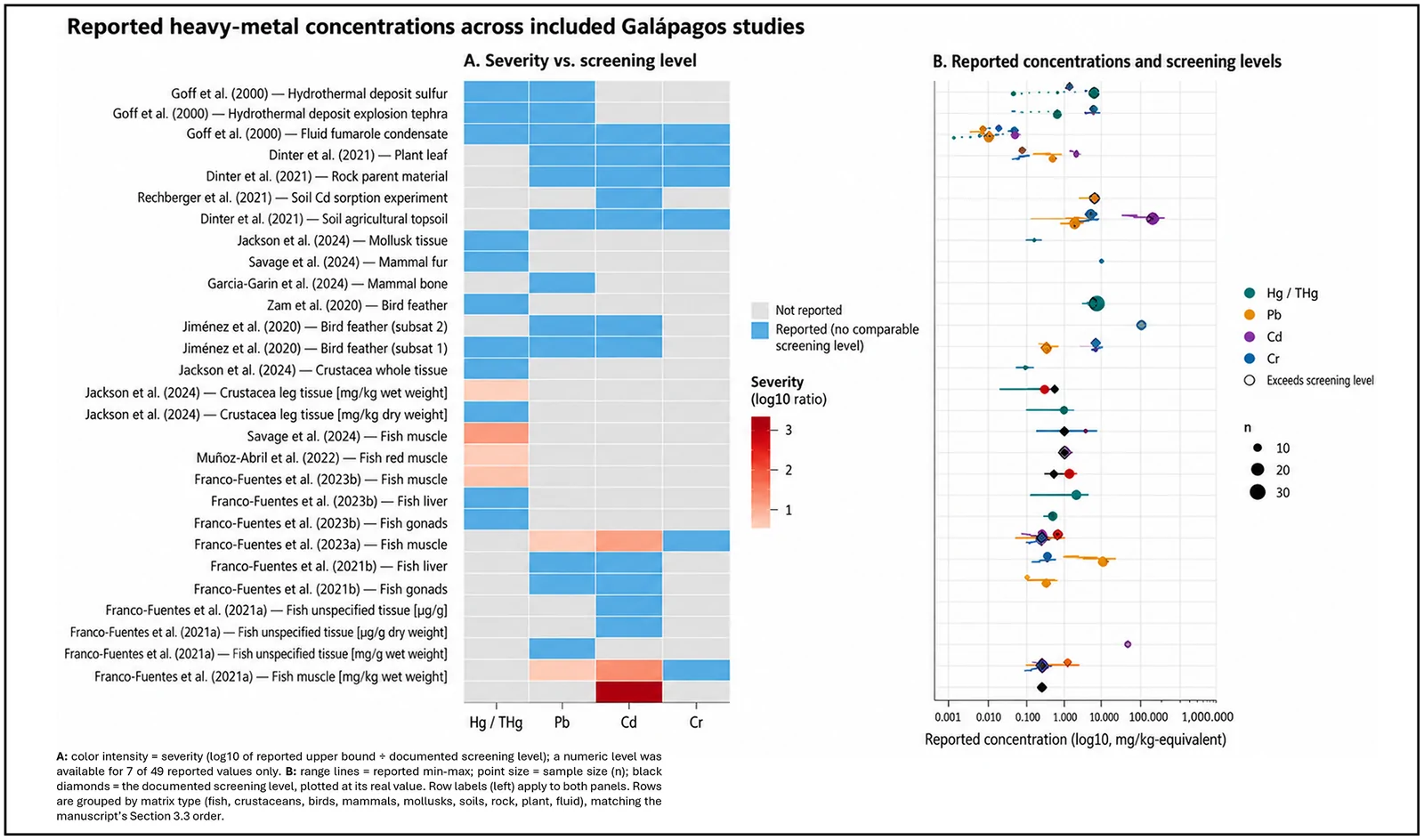
Reported heavy-metal concentrations across included Galápagos studies, relative to international screening levels. **(A)** Heatmap of reported values by source, sample category, and metal (Hg/THg, Pb, Cd, Cr). Color intensity reflects severity, defined as the reported upper-bound concentration divided by the applicable international screening/permissible level, on a log10 scale; a numeric screening level was identified for 9 of 49 reported values. Blue tiles indicate a reported value for which no comparable screening level was identified for that exact matrix and unit—this does not imply the value is within safe limits. Gray tiles indicate that metal was not reported for that source and sample category. **(B)** The same evidence expressed quantitatively: horizontal lines show the minimum–maximum reported concentration (log10 scale, mg/kg-equivalent) as originally published; point size reflects sample size (*n*); black diamonds mark the documented screening level at its true numeric position, where one exists. Row labels (shown once, left) apply to both panels and are grouped by matrix type, consistent with Supplementary Table S3 and Section 3.3.

### 2.6 Methodological quality assessment

Although methodological quality assessment is not a mandatory component of scoping reviews according to the JBI methodology, we deliberately incorporated an exploratory critical appraisal to strengthen the interpretation of the mapped evidence. Given the environmental and One Health focus of this review, evaluating the methodological robustness of the included studies allowed us to contextualize the consistency of reported findings, identify methodological strengths and weaknesses across different study designs, and highlight priorities for future research. Importantly, the quality assessment was not used as an eligibility criterion or to exclude studies, but solely to support evidence synthesis and interpretation.

Studies focused on human health were evaluated using the NIH Quality Assessment Tool. Environmental exposure studies were assessed with ROBINS-E, whereas experimental toxicological studies were evaluated using the ToxRTool according to their respective methodological designs.

### 2.7 Ethical considerations

This research did not require approval from a human research ethics committee, as it was based on secondary data sources. Nevertheless, emphasis was placed on ensuring that the included studies adhered to the minimum ethical principles established in the Declaration of Helsinki.

## 3 Results

### 3.1 General characteristics of the studies

Sixteen studies published between 2000 and 2025 were included, assessing the presence, distribution, and effects of heavy metals and trace elements across different compartments of the Galápagos Archipelago (Figure 2).

**Figure 2:**
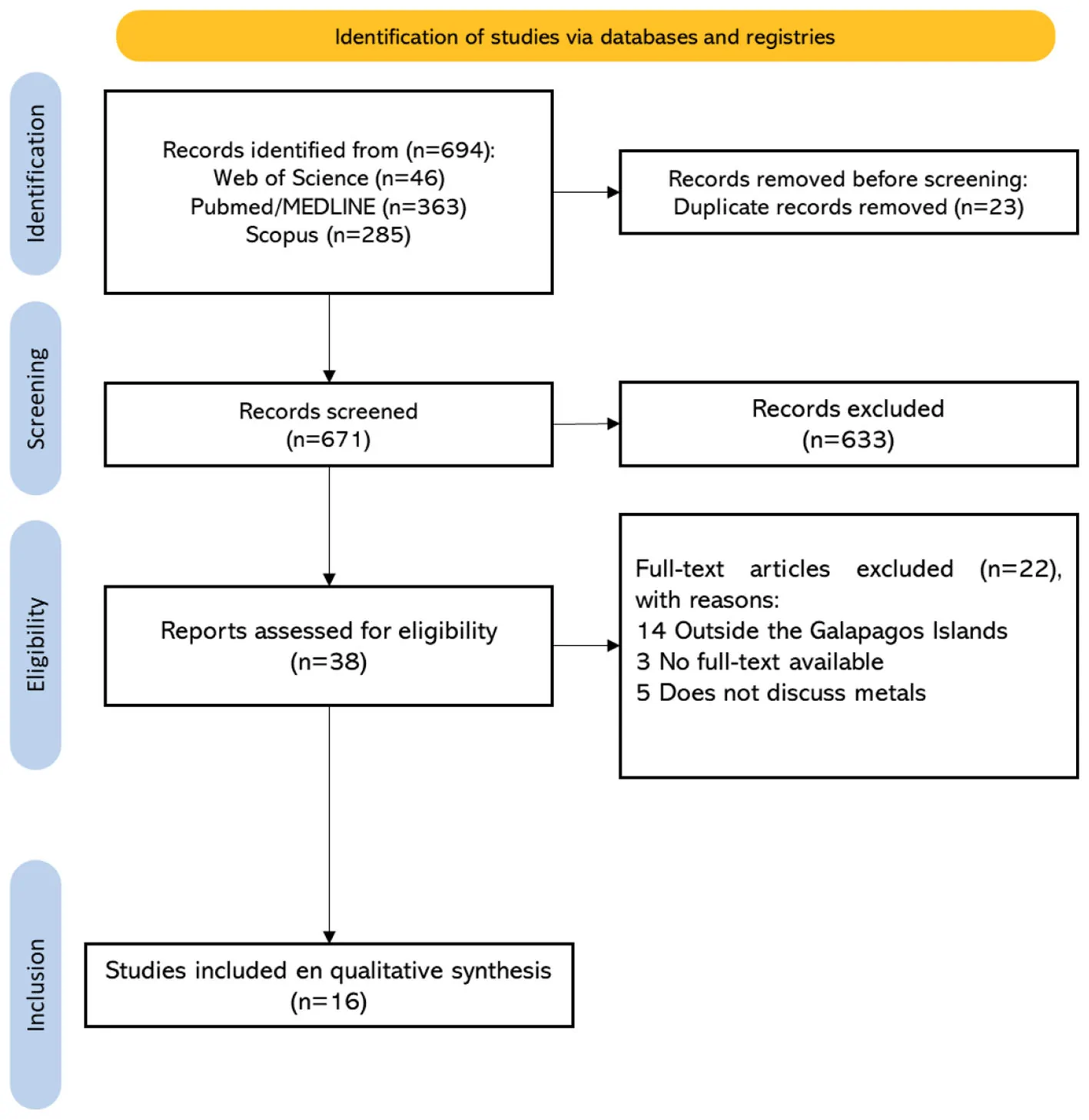
PRISMA flow diagram of the study selection process.

Table 1 summarizes the general characteristics of the included studies. Most studies were published between 2021 and 2025 (75.0%) and used observational analytical designs (68.7%). Marine fauna was the most frequently studied domain (50.0%), particularly fish (29.4%). Cadmium, mercury, and lead were the most commonly evaluated metals.

**Table 1:** Summary of included studies.

Characteristic	Category	*n* (%)
Publication period	2000–2010	1 (6.3%)
	2011–2020	3 (18.7%)
	2021–2025	12 (75.0%)
Study design	Analytical observational (cross-sectional)	11 (68.7%)
	Analytical observational (comparative)	2 (12.5%)
	Experimental/geochemical	1 (6.2%)
	Narrative/systematic review	2 (12.5%)
Main study domain	Marine fauna	8 (50.0%)
	Soil/terrestrial environment	4 (25.0%)
	Volcanic/hydrothermal environment	2 (12.5%)
	Multidomain environmental	2 (12.5%)
Type of matrix analyzed	Marine fish	5 (31.2%)
	Seabirds	2 (12.5%)
	Marine mammals	1 (6.2%)
	Crustaceans	1 (6.2%)
	Marine reptiles	1 (6.2%)
	Agricultural/volcanic soils	2 (12.5%)
	Volcanic/fumarolic deposits	2 (12.5%)
	Multiple matrices	2 (12.5%)
Most frequently evaluated metals^*^	Cadmium (Cd)	8 (50.0%)
	Mercury (Hg)	7 (43.7%)
	Lead (Pb)	7 (43.7%)
	Zinc (Zn)	6 (37.5%)
	Copper (Cu)	6 (37.5%)

^*^A single study may have evaluated multiple metals. *n*, number of studies; %, percentage; Cd, cadmium; Hg, mercury; Pb, lead; Zn, zinc; Cu, copper.

### 3.2 Critical appraisal

The methodological quality of the included studies was systematically evaluated using standardized critical appraisal tools. The ROBINS-E tool, the NIH Quality Assessment Tool, and the ToxRTool were applied according to the design and characteristics of each study. Overall, most studies assessed using ROBINS-E demonstrated clearly defined post-exposure assessment methods and objective outcome measurements (Figure 3). The primary source of potential bias was related to confounding, as inherent environmental variability could not be fully controlled in observational designs. Nevertheless, the consistency of findings across studies and the use of validated analytical techniques strengthen the overall reliability of the evidence. Only one article was evaluated using the NIH Quality Assessment Tool and was classified as having fair methodological quality, with strengths in clearly defined objectives and coherent conclusions (Figure 4). Finally, the toxicological studies evaluated using the ToxRTool demonstrated high methodological quality, with all studies classified as Category 1 (reliable without restrictions), indicating strong reliability and scientific coherence (Figure 5).

**Figure 3:**
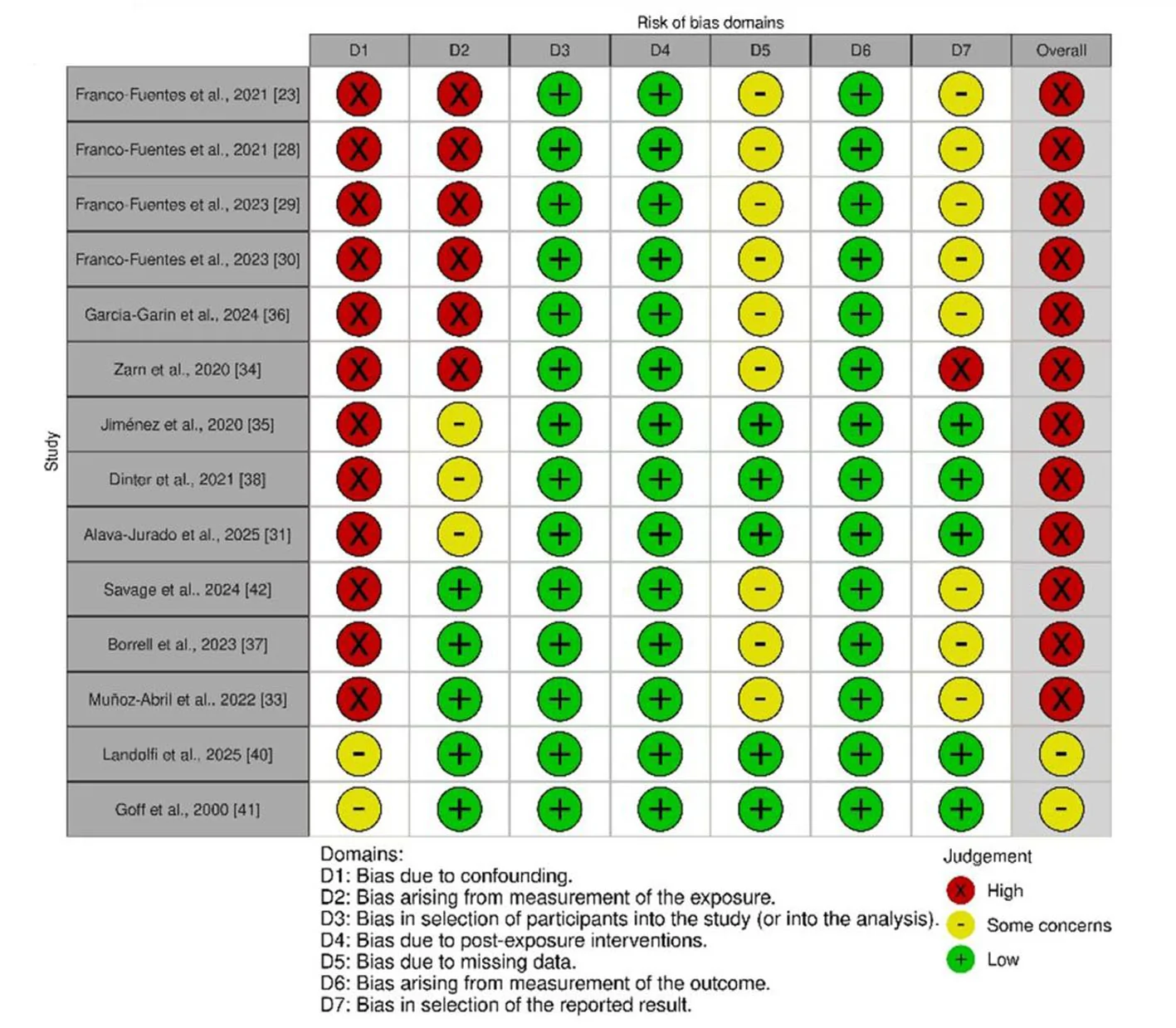
Risk of bias assessment using the ROBINS-E tool. The figure presents the domain-level and overall risk of bias judgments for all included studies. Individual study assessments across the seven ROBINS-E domains: bias due to confounding (D1), measurement of the exposure (D2), selection of participants (D3), post-exposure interventions (D4), missing data (D5), measurement of the outcome (D6), and selection of the reported result (D7), along with the overall risk of bias judgment.

**Figure 4:**
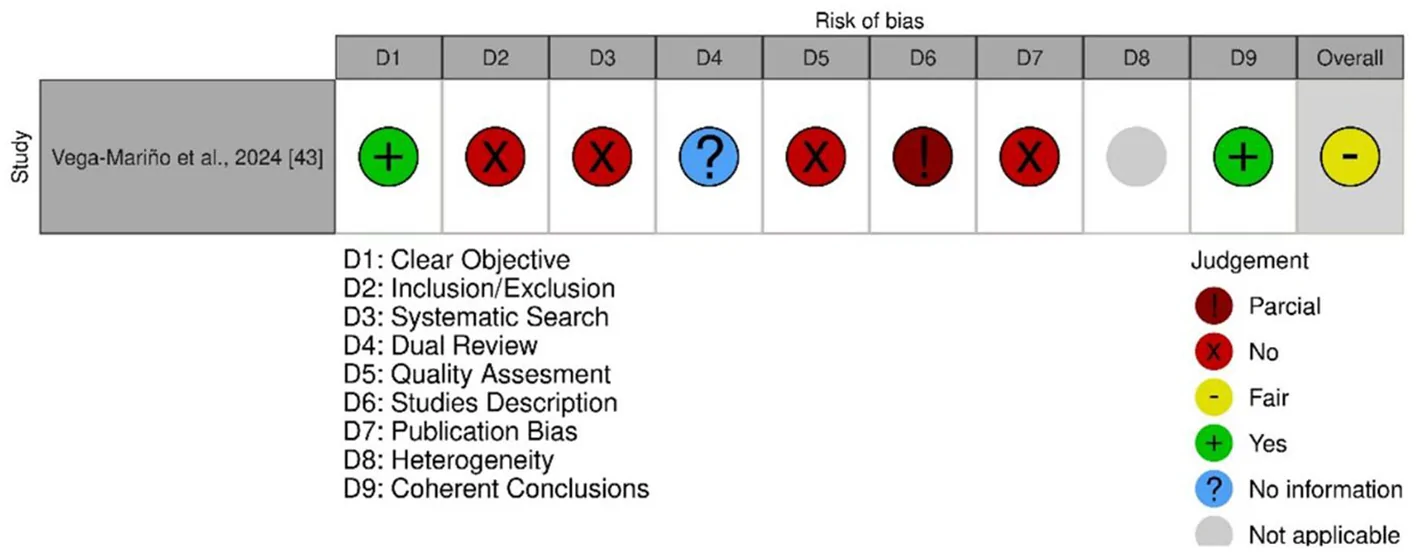
Methodological quality assessment using the National Institutes of Health (NIH) Quality Assessment Tool. The figure presents the domain-level and overall methodological quality evaluation of the included review across nine criteria: clear objective (D1), inclusion and exclusion criteria (D2), systematic search strategy (D3), use of dual review methods (D4), quality assessment of included studies (D5), adequate study description (D6), assessment of publication bias (D7), evaluation of heterogeneity (D8), and coherence of conclusions (D9). That shows the individual domain ratings, indicating strengths in clearly defined objectives and coherent conclusions, but important methodological limitations in several critical domains, including systematic search, quality assessment, and publication bias evaluation.

**Figure 5:**
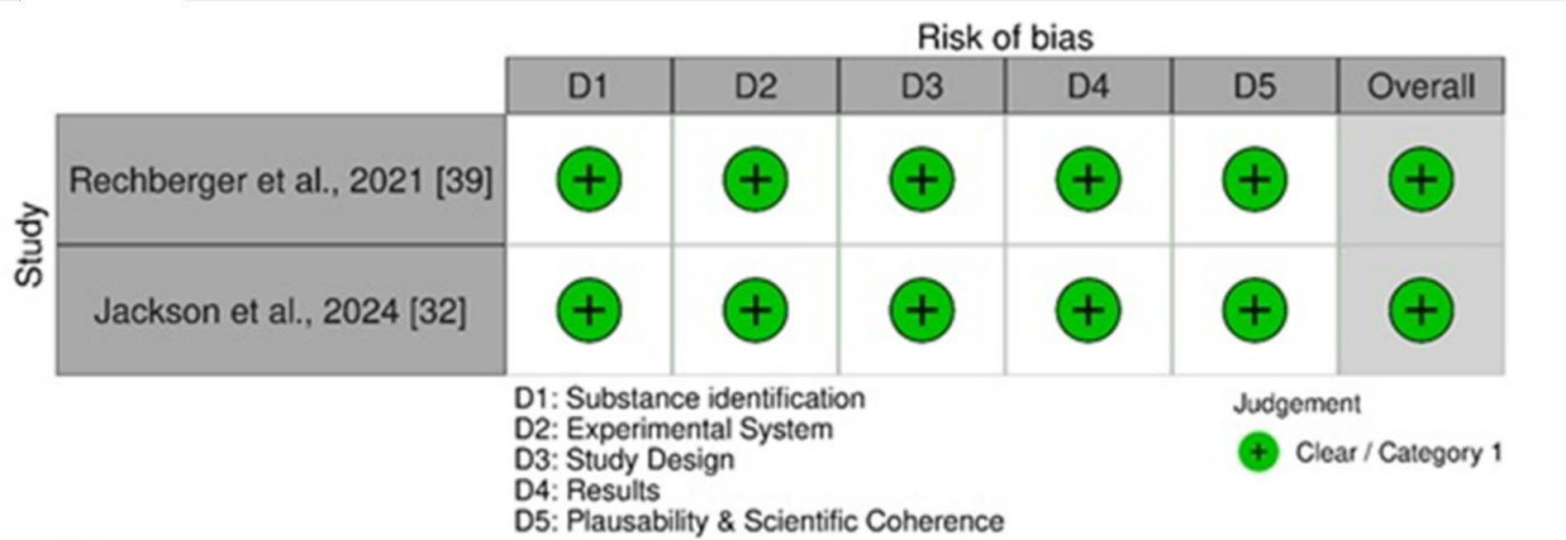
Risk of bias assessment using the ToxRTool. The figure presents the reliability evaluation of the included experimental studies across five domains: substance identification (D1), experimental system (D2), study design (D3), results documentation (D4), and plausibility and scientific coherence (D5), along with the overall reliability classification. The figure shows the individual study assessments, indicating that all studies met the criteria for reliability without restrictions.

### 3.3 Synthesis of findings

Detailed characteristics of the included studies, including sampling locations, study designs, biological and environmental matrices, metals assessed, analytical methods, and study-specific outcomes, are provided in Supplementary Table S3. Overall, the available evidence demonstrates substantial methodological and environmental heterogeneity, reflecting the diversity of matrices, species, geographic locations, and analytical approaches evaluated across the Galápagos Archipelago. To facilitate interpretation, the following synthesis focuses on the main patterns of heavy metal occurrence, bioaccumulation, potential sources, and toxicological and ecological implications identified across the included studies.

Overall, the studies encompassed multiple matrices, primarily marine fauna, volcanic and agricultural soils, as well as hydrothermal systems and mineral deposits associated with volcanic activity. The metals evaluated mainly included cadmium, mercury, lead, zinc, copper, iron, aluminum, and other trace elements, which were quantified using analytical techniques such as inductively coupled plasma mass spectrometry (ICP-MS), optical emission spectrometry (ICP-OES), direct mercury analyzers (DMA), and atomic absorption spectrometry.

To visualize the spatial distribution of heavy-metal research across the Galápagos Archipelago, a geostatistical interpolation (kriging-like approach) was applied using reported sampling locations extracted from the included studies. Research intensity was stratified by matrix (fish, birds, and soils) to identify spatial clustering patterns and potential research hotspots. The resulting spatial surface illustrates heterogeneous research coverage, with a notable concentration around central and southeastern islands, particularly Santa Cruz, and comparatively lower intensity in northern and more remote islands (Figure 6). These maps do not represent measured contamination levels but rather the spatial distribution of sampling locations reported in the included studies, allowing visualization of areas with greater or lower research coverage.

**Figure 6:**
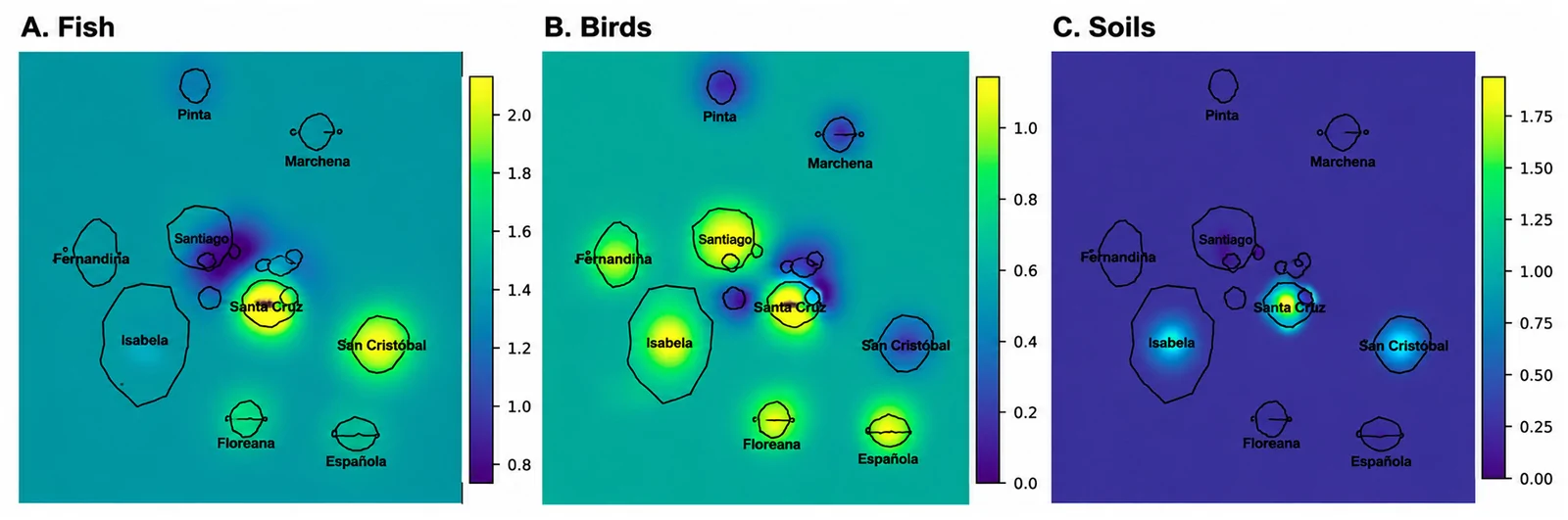
Spatial distribution of heavy-metal research across the Galápagos Archipelago. Geostatistical interpolation (kriging-like approach based on Gaussian process regression) was applied to the geographic sampling locations extracted from the studies included in this scoping review to generate continuous spatial surfaces representing the relative spatial distribution of research effort. Panels display research coverage stratified by matrix (fish, birds, and soils). Warmer colors indicate areas with a higher relative density of sampling locations reported in the included studies, whereas cooler tones indicate lower research coverage. Island outlines are shown for geographic reference. These interpolated surfaces do not represent measured heavy metal concentrations or environmental contamination intensity; rather, they provide an evidence map illustrating the spatial distribution of the available research across the Galápagos Archipelago. The figure highlights spatial heterogeneity in research effort, with greater concentration in the central and southeastern islands, particularly Santa Cruz. Panels display research coverage stratified by matrix: **(A)** fish, **(B)** birds, and **(C)** soils.

#### 3.3.1 Heavy metals in marine fish

In an assessment of commercial fish species from the Galápagos Marine Reserve, cadmium, lead, zinc, copper and chromium were identified in muscle tissue, with demersal species showing higher concentrations than pelagic species (34). Cadmium accumulation has also been documented in the liver of demersal fish, particularly *Caulolatilus spp*. and *Paralabrax albomaculatus*, with redistribution of zinc toward gonadal tissue during reproductive processes (39). Franco-Fuentes et al. (40) further reported that cadmium concentrations exceeded EFSA maximum permissible limits across multiple commercial fish species, with *Caulolatilus affinis* presenting the highest toxicological risk; detectable concentrations of lead, zinc, and copper were also identified.

Regarding mercury, Franco-Fuentes et al. (19) detected this metal in muscle, hepatic, and gonadal tissues, with concentrations varying across species and tissue types. Additionally, Alava-Jurado et al. (41) identified barium, manganese, magnesium, and strontium in the otoliths of reef fish, with significant differences among sampling sites.

#### 3.3.2 Heavy metals in crustaceans, reptiles, birds, and marine mammals

Jackson et al. detected mercury in muscle tissue of the crab *Grapsus grapsus* across multiple sites in the archipelago, with differences in concentrations between locations (42). Muñoz-Abril et al. (43) reported total mercury concentrations of 0.20–9.60 mg/kg wet weight in yellowfin tuna (*Thunnus albacares*) from the Galápagos Marine Reserve, showing that mercury accumulation was primarily influenced by regional food-web dynamics and oceanographic processes, while Jiménez et al. (44) identified the presence of lead and cadmium in feathers of aquatic birds. In marine mammals, Garcia-Garin et al. (45) detected lead in bone tissue of *Zalophus wollebaeki*.

#### 3.3.3 Heavy metals in volcanic and agricultural soils

In terrestrial matrices, Dinter et al. (46) detected multiple heavy metals—including cadmium, cobalt, chromium, copper, nickel, lead, and zinc—in agricultural soils from the islands of Santa Cruz, San Cristóbal, and Isabela. Consistently, Rechberger et al. (47) identified cadmium in volcanic soils across different stages of pedogenic development.

In active volcanic environments, Landolfi et al. (48) detected trace metals—including aluminum, iron, zinc, manganese, and copper—in mineral encrustations associated with fumaroles of the Sierra Negra volcano. Likewise, Goff et al. (49) reported the presence of trace metals, such as zinc and copper, in volcanic hydrothermal systems, including associated fluids and mineral deposits.

#### 3.3.4 Environmental distribution of heavy metals

The distribution of heavy metals in the Galápagos Archipelago has been documented across multiple environmental and biological matrices, including marine fauna, soils, sediments, and volcanic systems. In this context, Savage et al. (50) conducted a comprehensive assessment of the chemical landscape of the Galápagos Marine Reserve, identifying the presence of heavy metals such as mercury, lead, cadmium, zinc, copper, and chromium in diverse environmental matrices and marine organisms. These metals were detected in fish, seabirds, marine mammals, sediments, and soils, demonstrating their widespread distribution across both marine and terrestrial ecosystems of the archipelago. The study also reported spatial variability in metal occurrence, particularly in coastal zones and areas associated with volcanic activity and human settlements.

Complementarily, Vega-Mariño et al. (51), documented the presence of multiple heavy metals across different ecosystem components, including agricultural soils, marine fauna, and environmental matrices associated with both terrestrial and marine ecosystems. This study reported the detection of metals such as cadmium, copper, nickel, zinc, and chromium in volcanic and agricultural soils, as well as the presence of heavy metals in fish and other marine species. Moreover, the study integrated evidence from various investigations conducted within the archipelago, confirming the occurrence of heavy metals across multiple ecological levels and environmental matrices.

To synthesize the quantitative evidence across included studies, reported concentrations of the most frequently assessed metals (Hg/THg, Pb, Cd, and Cr) were compiled by source and sample category and compared against applicable international screening levels (Table 2). Of 49 extracted concentration values, 9 exceeded a documented screening level, concentrated in fish muscle (cadmium, lead, mercury), red muscle in yellowfin tuna, and select crustacean tissue. The remaining values could not be directly compared against a regulatory threshold, either because no matrix-specific level exists or because the reported tissue or unit basis differed from standard guideline categories, a gap discussed further in Section 4.3.

**Table 2:** Quantitative synthesis of reported heavy-metal concentrations relative to international screening levels.

Summary table: reported Galápagos heavy-metal values by source and sample category								
**Evidence map/reported-value range table. Unit/basis is row-specific to prevent invalid cross-matrix comparisons**.								
Source/citation	Sample category	Unit/basis	Hg/THg	Pb	Cd	Cr	Records summarized	Species/sites summarized
Zarn et al. (74) — Marine Ornithology 48:71–80	Biota | Bird | feather	ppm	Hg: 3.7–14.51 (*n* = 38)	—	—	—	38	8 species/taxa
Jiménez et al. (44) — Marine Ornithology 48:85–89	Biota | Bird | feather	ppm	Hg: < DL (*n* = 17)	7.085–11.16 (*n* = 9)	0.16–0.479 (*n* = 9)	—	35	4 species/taxa
Jiménez et al. (44) — Marine Ornithology 48:85–89	Biota | Bird | feather	ppm	—	< DL−193 (*n* = 7; incl. < DL)	< DL (*n* = 7)	—	14	3 species/taxa
Jackson et al. (42) — Fishes 9:233	Biota | Crustacea | leg tissue	mg/kg dry weight	Hg: 0.06–2.04 (*n* = 5)	—	—	—	5	1 species/taxa
Jackson et al. (42) — Fishes 9:233	Biota | Crustacea | leg tissue	mg/kg wet weight	Hg: 0.0144–**0.51**^*a*^ (*n* = 5)	—	—	—	5	1 species/taxa
Jackson et al. (42) — Fishes 9:233	Biota | Crustacea | whole tissue	mg/kg dry weight	Hg: 0.0449–0.1212 (*n* = 2)	—	—	—	2	1 species/taxa
Franco-Fuentes et al. (39) — Marine Pollution Bulletin 173:112953	Biota | Fish | gonads	mg/kg wet weight (w.w.)	—	0.1 (*n* = 2)	0.1–0.6 (*n* = 3)	—	5	3 species/taxa
Franco-Fuentes et al. (19) — Journal of Food Composition and Analysis 115:104969	Biota | Fish | gonads	mg/kg wet weight	Hg: < DL−0.49 (*n* = 8; incl. < DL)	—	—	—	8	8 species/taxa
Franco-Fuentes et al. (39) — Marine Pollution Bulletin 173:112953	Biota | Fish | liver	mg/kg wet weight (w.w.)	—	0.1–0.6 (*n* = 5)	0.9–23.1 (*n* = 5)	—	10	6 species/taxa
Franco-Fuentes et al. (19) — Journal of Food Composition and Analysis 115:104969	Biota | Fish | liver	mg/kg wet weight	Hg: 0.12–4.24 (*n* = 9)	—	—	—	9	9 species/taxa
Franco-Fuentes et al. (34) — Journal of Environmental Management 286:112188	Biota | Fish | muscle	mg/kg fresh weight	—	0.077–**0.443**^*b*^ (*n* = 4)	0.097–2.543^*c*^ (*n* = 4)	0.138–0.409 (*n* = 4)	12	4 species/taxa
Franco-Fuentes et al. (34) — Journal of Environmental Management 286:112188	Biota | Fish | muscle	mg/kg wet weight	—	—	**2.4** ^*d*^	—	1	1 species/taxa
Franco-Fuentes et al. (40) — Marine Pollution Bulletin 189:114739	Biota | Fish | muscle	mg/kg wet weight	—	0.09–**0.44**^*b*^ (*n* = 5)	0.05–**1.24**^*c*^ (*n* = 5)	0.06–0.34 (*n* = 5)	15	5 species/taxa
Franco-Fuentes et al. (19) — Journal of Food Composition and Analysis 115:104969	Biota | Fish | muscle	mg/kg wet weight	Hg: < DL–**1.26**^*e*^ (*n* = 9; incl. < DL)	—	—	—	9	9 species/taxa
Savage et al. (50) — Science of the Total Environment 954:176659 [secondary review]	Biota | Fish | muscle	mg/kg wet weight	THg: 0.18–**8.29**^*f*^ (*n* = 2)	—	—	—	2	2 species/taxa
Muñoz-Abril et al. (43) — Environmental Toxicology and Chemistry 41:2732–2744	Biota | Fish | red muscle	ppm wet weight	THg: 0.71–**1.72**^*g*^ (*n* = 5)	—	—	—	5	1 species/taxa
Franco-Fuentes et al. (34) — Journal of Environmental Management 286:112188	Biota | Fish | unspecified tissue	μg/g	—	—	6	—	1	1 species/taxa
Franco-Fuentes et al. (34) — Journal of Environmental Management 286:112188	Biota | Fish | unspecified tissue	μg/g dry weight	—	—	2	—	1	1 species/taxa
Franco-Fuentes et al. (34) — Journal of Environmental Management 286:112188	Biota | Fish | unspecified tissue	mg/kg wet weight	—	0.06–0.07 (*n* = 2)	—	—	1	1 species/taxa
Garcia-Garin et al. (45) — Estuarine, Coastal and Shelf Science 297:108614	Biota | Mammal | bone	mg/kg dry weight	—	0.12	—	—	1	1 species/taxa
Savage et al. (50) — Science of the Total Environment 954:176659 [secondary review]	Biota | Mammal | fur	mg/kg dry weight	THg: 15.41–16.1 (*n* = 2)	—	—	—	2	2 species/taxa
Jackson et al. (42) — Fishes 9:233	Biota | Mollusk | tissue	mg/kg dry weight	Hg: 0.07–0.2284 (*n* = 2)	—	—	—	2	1 species/taxa
Goff et al. (49) — Bulletin of Volcanology 62:34–52	Environment | Fluid | fumarole condensate	ppm	Hg: < 0.0002–0.048 (*n* = 9; incl. < DL)	< 0.002–0.013 (*n* = 9; incl. < DL)	< 0.001–0.006 (*n* = 9; incl. < DL)	< 0.002–0.048 (*n* = 9; incl. < DL)	36	environmental/site-level values
Goff et al. (49) — Bulletin of Volcanology 62:34–52	Environment | Hydrothermal deposit | explosion tephra	ppm	Hg: < 0.02–1.1 (*n* = 6; incl. < DL)	4.7–12 (*n* = 6)	—	—	12	environmental/site-level values
Goff et al. (49) — Bulletin of Volcanology 62:34–52	Environment | Hydrothermal deposit | sulfur	ppm	Hg: < 0.02–16.2 (*n* = 10; incl. < DL)	< 1–2 (*n* = 10; incl. < DL)	—	—	20	environmental/site-level values
Dinter et al. (46) — Chemosphere 272:129821	Environment | Plant | leaf	mg/kg	—	0.02–0.07 (*n* = 3)	0.12–0.73 (*n* = 3)	1.46–3.47 (*n* = 3)	9	3 species/taxa
Dinter et al. (46) — Chemosphere 272:129821	Environment | Rock | parent material	mg/kg	—	0.67	0.09	111	3	environmental/site-level values
Dinter et al. (46) — Chemosphere 272:129821	Environment | Soil | agricultural topsoil	mg/kg	—	0.855–11.6 (*n* = 19)	0.078–4.11 (*n* = 19)	45.3–648 (*n* = 19)	57	environmental/site-level values
Rechberger et al. (47) — Journal of Environmental Quality 50:1233–1245	Environment | Soil | Cd sorption experiment	mg/kg soil	—	—	3.5–12.2 (*n* = 14)	—	14	1 species/taxa

Cells show min–max ranges of reported central/statistical values from the master table; no pooling was performed across matrices, tissues, wet/dry basis or units.

Included metals: Hg/THg, MeHg, Pb, Cd and Cr. Excluded from this summary: non-heavy metals, isotope signatures, percentages, risk-intake calculations and non-concentration metrics.

**Permissible-level markers:**

Superscript numerals and bold letters indicate ranges whose upper value exceeds the selected permissible/screening level for the corresponding sample category and unit/basis. Unmarked cells were either not directly comparable with an available level or did not exceed the selected reference.

^*a*^ Hg, crustacean leg tissue, wet weight — EU 2023/915 maximum level (ML): 0.50 mg/kg wet weight for crustaceans/molluscs/fish muscle; upper bound 0.51 mg/kg exceeds the ML.

^*b*^ Pb, fish muscle, wet/fresh weight — Codex CXS 193-1995/EU 2023/915 ML: 0.30 mg/kg wet weight for fish muscle; upper bound exceeds the ML.

^*c*^ Cd, fish muscle, wet/fresh weight — EU 2023/915 ML range for fish: 0.050–0.25 mg/kg wet weight, depending on species; upper bound exceeds the upper fish ML.

^*d*^ Cd, fish muscle, mg/g wet weight — EU 2023/915 fish Cd ML range: 0.050–0.25 mg/kg wet weight; reported value 2.4 mg/g wet weight ( =2,400 mg/kg) exceeds the ML range.

^*e*^ Hg, fish muscle, wet weight — EU 2023/915 ML: 0.50 mg/kg wet weight for fish muscle/general fishery products; upper bound exceeds the ML. Species-specific exceptions may apply.

^*f*^ THg, shark muscle, wet weight — EU 2023/915 Hg ML used as screening reference; for shark/large predatory fish the higher 1.0 mg/kg category can apply, but the upper bound 8.29 mg/kg exceeds both 0.50 and 1.0 mg/kg.

^*g*^ THg, tuna red muscle, wet weight — EU 2023/915 Hg ML used as screening reference; for tuna-like predatory fish the higher 1.0 mg/kg category can apply. Upper bound 1.72 mg/kg exceeds 1.0 mg/kg.

Concentrations are expressed as mg/kg (wet or dry weight, as reported in the original source); values reported in mg/g were converted to mg/kg-equivalent for comparability across studies. Basis (wet weight, dry weight, or fresh weight) is preserved per row, as these are not directly interchangeable. ML = maximum level, per Codex Alimentarius / EU Regulation 2023/915, applied where a matrix- and species-comparable threshold exists. “ < ” indicates a value at or below the analytical detection limit; ranges beginning with “ < ” indicate the lower bound was below detection while the upper bound was quantified. ND = all reported values were below detection.

To complement the tabulated values with a visual overview, the same evidence is presented graphically in Figure 1. The figure combines a heatmap of severity relative to documented screening levels (panel A) with the underlying quantitative concentration ranges (panel B), allowing patterns across matrices and sources to be assessed briefly alongside the precise reported values and sample sizes summarized in Supplementary Table S3.

## 4 Discussion

The findings of this review confirm the heterogeneous presence of heavy metals across multiple environmental and biological compartments of the archipelago, which can be characterized as a complex biological and ecological phenomenon. Environmental contamination is exacerbated by industrial development and anthropogenic activities. The continuous and substantial discharge of pollutants into the oceans generates toxicological hazards in coastal environments; however, contamination is not confined to marine areas, as heavy metals are also present within our most immediate surroundings (52). The Galápagos Islands represent a hybrid scenario in which diverse natural sources and human activities converge within a dynamic socioecological system that must be examined through a One Health conceptual framework to ensure coherence in understanding the interconnected impacts on human and animal health (51).

Heavy metals enter trophic chains and are progressively transferred to higher trophic levels, ultimately creating potential pathways for human exposure through the consumption of contaminated marine organisms. The evidence synthesized in this review demonstrates processes of bioaccumulation and biomagnification across different marine trophic levels. Demersal species (those inhabiting the seafloor) exhibit higher concentrations of cadmium and mercury compared to pelagic species (which inhabit the water column) (32, 34, 40), consistent with their greater contact with metal-enriched volcanic sediments. Marine fauna—the primary focus of our study—is also examined by Liu et al. (31), who reported that bioaccumulation of heavy metals varies significantly among marine species depending on habitat, feeding patterns, and trophic level. A substantial portion of this fauna, whether more or less contaminated, is ultimately consumed by humans, representing a plausible route of dietary exposure. Based on evidence from the broader scientific literature, heavy metal accumulation in marine organisms may reduce essential nutrient content and has been associated with adverse health outcomes in humans, including impaired immune function, intrauterine growth restriction, deterioration of psychosocial behavior, nutrition-related disabilities, and an increased risk of upper gastrointestinal cancers (53). However, these health outcomes have not been directly evaluated in the Galápagos population, as no human biomonitoring or epidemiological studies were identified in this review.

Volcanic and hydrothermal activity constitutes a significant source of trace metals such as iron, zinc, copper, and mercury, as evidenced in fumarolic deposits and hydrothermal systems associated with structural faults (48, 49). In other active volcanic archipelagos, such as *Kilauea* in Hawaii, volcanic emissions have been shown to significantly increase atmospheric concentrations of heavy metals to levels comparable to those of industrialized cities (54). A biomonitoring study conducted in populations residing near Mount Etna documented significantly higher urinary concentrations of arsenic, mercury, lead, and other metals among individuals living in closer proximity to volcanic deposits (55). These findings suggest that volcanic activity may represent a plausible source of chronic environmental exposure; however, this hypothesis has not yet been confirmed through human biomonitoring studies conducted in the Galápagos population.

Concurrently, agricultural soils have been shown to contain cadmium, copper, zinc, and other metals, with patterns suggesting anthropogenic influence linked to phosphate fertilizers (46), alongside increasing mobility of cadmium in more developed volcanic soils (47). These studies support the findings of the present research, in which metals such as cadmium, cobalt, chromium, copper, nickel, lead, and zinc were identified in agricultural soils from the islands of Santa Cruz, San Cristóbal, and Isabela. As noted by Rashid et al. (56), heavy metals are non-degradable elements, unlike certain organic toxicants, and tend to persist in the environment over long periods. Based on evidence from studies conducted outside the Galápagos Archipelago, chronic dietary exposure to these metals has been associated with adverse outcomes, including cardiovascular disease, neurodegenerative disorders, skeletal alterations, and several cancers (56, 57). However, these associations have not yet been evaluated in the Galápagos population. Nevertheless, evidence from studies conducted outside the Galápagos Archipelago suggests that chronic dietary exposure to these metals may contribute to an increased risk of the formentioned adverse health outcomes, particularly cancers of the digestive system, skin, and lungs (1, 58).

Although no primary studies involving human populations or human biomonitoring were identified, several investigations reported that commercial fish species from the Galápagos exceeded international regulatory limits for cadmium and mercury established by EFSA and FDA (19, 40). Given that the local population maintains a dietary pattern characterized by high fish consumption, these findings suggest a plausible pathway of chronic dietary exposure. Consequently, the human health dimension of the present One Health synthesis should be interpreted as a probable exposure pathway inferred from environmental and wildlife evidence rather than as a component directly supported by human epidemiological or biomonitoring data. Therefore, the health consequences of this potential exposure remain unknown, and this lack of primary human evidence represents one of the most important knowledge gaps identified in this review. The archipelago's wildlife therefore acts simultaneously as a victim of contamination and as a sentinel of potential human exposure, highlighting the need for future human biomonitoring and epidemiological studies.

### 4.1 Heavy metal exposure

The environmental and toxicological relevance of potentially toxic elements depends not only on their concentrations but also on their chemical speciation, bioavailability, and trophic behavior. Hg is of particular concern because microbial methylation produces methylmercury, a highly bioavailable form that bioaccumulates in aquatic organisms and biomagnifies through marine food webs. In contrast, As, Cd, Pb, Cu, and Zn exhibit more variable patterns of accumulation and trophic transfer, depending on environmental conditions, organism physiology, and food-web structure (1–3). Despite these differences, exposure to these elements may involve common cellular mechanisms, including oxidative stress, depletion of antioxidant defenses, mitochondrial dysfunction, enzyme inhibition, protein alterations, and DNA damage, potentially contributing to neurological, renal, hepatic, cardiovascular, genotoxic, and carcinogenic effects (17, 18).

In the present review, methylmercury, cadmium, lead, and copper were the elements most frequently investigated in the scientific literature on the Galápagos Archipelago. The following sections summarize their principal toxicological effects based on the broader scientific literature to contextualize the potential health implications of the environmental contamination documented in the archipelago. The following sections summarize their principal toxicological effects based on the broader scientific literature to contextualize the potential health implications of the environmental contamination documented in the archipelago.

#### 4.1.1 Methylmercury

MeHg contamination emerged as a relevant concern in the Galápagos marine ecosystem, particularly because elevated concentrations were identified in species that may contribute to trophic transfer and human dietary exposure. Franco-Fuentes et al. (19) reported that three commercially important fish species, *Acanthocybium solandri, Pontinus clemensi*, and *Hyporthodus mystacinus*, exceeded international regulatory limits established by EFSA, FDA, and Codex for mercury in muscle tissue. Notably, the highest concentration was observed in *H. mystacinus* (1.26 mg/kg wet weight), while demersal species showed higher estimated weekly Hg intakes than pelagic species (19). These findings are particularly relevant in the Galápagos context because fish constitute an important source of animal protein for the local population.

Evidence from other components of the Galápagos marine food web reinforces this concern. Jackson et al. (42) found marked spatial variation in total mercury concentrations in *Grapsus grapsus*, with the highest levels occurring at San Cristóbal and South Plaza. All crabs sampled at San Cristóbal and 50% of those from South Plaza exceeded the US EPA threshold used by the authors for seafood consumption (42). Importantly, elevated concentrations were not restricted to areas with greater human influence, as South Plaza, a protected site, also showed unexpectedly high Hg concentrations. This pattern suggests that mercury occurrence in the archipelago cannot be attributed exclusively to local anthropogenic activity. Volcanic inputs have been proposed as a plausible natural source, although localized anthropogenic contributions and trophic differences in diet cannot be excluded (19, 42).

The ecological and public health relevance of these findings is strengthened by the behavior of mercury within marine food webs. MeHg constitutes a major fraction of mercury accumulated in fish and is efficiently transferred and biomagnified across trophic levels (59, 60). Consequently, Hg detected in commercial fish and intertidal invertebrates should be interpreted not only as evidence of environmental contamination but also as an indicator of potential trophic exposure within the Galápagos ecosystem. This is particularly relevant because MeHg is a potent neurotoxicant that readily crosses the blood–brain and placental barriers, with fetal and early-life development representing especially susceptible periods (60, 61). Experimental evidence has also reported persistent impairments in motor function, coordination, and memory following perinatal MeHg exposure (62). However, the corresponding health implications for Galápagos residents remain to be established locally.

#### 4.1.2 Cadmium

Cd contamination in the Galápagos is particularly relevant because evidence was identified in both marine and terrestrial food-related matrices, suggesting multiple potential pathways of exposure. In commercial fish, Franco-Fuentes et al. (40) reported that all evaluated species exceeded the maximum allowable concentration for Cd established by EFSA. Tissue-specific analyses further showed high Cd concentrations in the liver of the demersal species *Caulolatilus princeps, Caulolatilus affinis*, and *Paralabrax albomaculatus*, whereas comparable concentrations were not observed in the evaluated pelagic species (39). The authors proposed that the greater accumulation in demersal fish could be related to their closer interaction with the seafloor and to the volcanic origin of metals in the archipelago (39).

The terrestrial evidence indicates a complementary pathway of concern. Agricultural soils from Isabela, Santa Cruz, and San Cristóbal contained Cd concentrations that were high relative to many other locations worldwide, while Cd mobility increased with soil age and decreasing pH (46). The same study suggested that Cd enrichment, particularly on Santa Cruz, may partly reflect the historical use of phosphorus fertilizers and demonstrated that Cd in an affected agricultural site was readily available to plants (46). Consistently, Rechberger et al. (47) showed that Cd retention decreases substantially as Galápagos volcanic soils age, with up to 60% of added Cd remaining only weakly sorbed in highly weathered soils, thereby increasing its potential mobility and transfer into the food chain. Together, these findings indicate that Cd in the Galápagos should not be considered solely a marine contaminant but rather a One Health concern spanning marine resources, agricultural soils, and potentially locally produced foods.

This environmental pattern is toxicologically relevant because Cd can enter cells through transport systems normally used by essential divalent elements, including calcium- and zinc-related pathways (63, 64), and chronic exposure is characterized by progressive body accumulation, particularly in the kidney (1, 61). Experimental evidence supports Cd uptake through voltage-sensitive calcium channels (63), while ZIP8-mediated transport demonstrates competition between Cd^2+^ and Zn^2+^ (64). From a population-health perspective, this persistence is important because prolonged dietary exposure may be more consequential than occasional consumption of foods with high Cd concentrations (46). Epidemiological evidence has also associated Cd exposure with cardiovascular disease, particularly coronary heart disease, in addition to its established renal and skeletal toxicity (1, 61, 65, 66). However, whether the combined marine and terrestrial contamination pathways identified in the Galápagos translate into measurable Cd exposure or health effects among residents remains to be established locally.

#### 4.1.3 Lead

Pb contamination in the Galápagos appears to follow a more heterogeneous and comparatively limited pattern than that observed for mercury or cadmium. Garcia-Garin et al. (45) detected Pb in the bones of *Zalophus wollebaeki*, but concentrations were significantly lower than those reported in pinniped populations from Mauritania and Uruguay, supporting the characterization of the Galápagos as a comparatively unpolluted environment for this element (45). Because these pinniped populations are non-migratory and bone reflects long-term Pb accumulation, they may provide useful information on chronic environmental exposure within their respective marine habitats.

Nevertheless, the detection of Pb in other environmental and biological matrices indicates that the element is present within the archipelago. Jiménez et al. (44) identified Pb in feathers of Galápagos flamingos, although the authors could not attribute these concentrations to a specific anthropogenic or near-island source. Similarly, Pb concentrations in agricultural soils were generally low, although Dinter et al. (46) suggested that some enrichment may occur through phosphorus fertilizers and soil retention processes (46). These findings suggest that Pb exposure in the Galápagos is spatially and environmentally variable rather than indicative of widespread contamination. This interpretation is consistent with broader evidence showing that local anthropogenic inputs of heavy metals are likely concentrated around inhabited areas and harbors, although current monitoring remains insufficient to define contaminant-specific spatial patterns (42, 50).

From a public health perspective, even comparatively low environmental Pb levels warrant attention because neurodevelopmental effects have been observed at low blood lead concentrations. In a pooled analysis of seven prospective cohorts, Lanphear et al. (67) reported a 6.9-point decrease in IQ across an increase in concurrent blood Pb concentrations from 2.4 to 30 μg/dL, with the steepest declines occurring below 10 μg/dL (67). This is particularly relevant for children, who are more susceptible to Pb-associated neurodevelopmental effects (1, 10, 60). Whether the documented environmental Pb contamination translates into measurable human exposure remains to be established locally.

#### 4.1.4 Copper

Cu was among the metals repeatedly identified across environmental matrices in the Galápagos, with particularly relevant findings in agricultural soils. Dinter et al. (46) reported that Cu concentrations in soils from Isabela, Santa Cruz, and San Cristóbal were high compared with many other locations worldwide. The authors also suggested that Cu enrichment, together with Cd and Zn, may partly reflect the historical use of agrochemicals, particularly on Santa Cruz Island. Nearly all analyzed soils exceeded the CCME soil quality guideline for Cu, while maximum concentrations across the studied islands also surpassed the corresponding MEF reference value (46).

The occurrence of Cu in Galápagos should nevertheless be interpreted within the particular geochemical context of a volcanic archipelago. Hydrothermal systems are present in active volcanic areas such as Sierra Negra and Alcedo, where fumarolic and hydrothermal activity can mobilize and redistribute trace elements through volcanic gases, altered rocks, and mineral deposits (49). More recent observations from Sierra Negra have also shown that Cu forms part of the chemical composition of fumarolic mineral incrustations, reinforcing the contribution of natural geochemical processes to the local distribution of this element (48). Therefore, the Cu pattern observed in the archipelago likely reflects an interaction between natural volcanic inputs and anthropogenic activities, rather than a single contamination source.

From a One Health perspective, this distinction is important because agricultural soils may represent a link between environmental contamination and locally produced food, while Cu detected in other environmental and biological matrices indicates broader ecosystem circulation. However, Cu is also an essential micronutrient subject to tightly regulated physiological homeostasis, and toxicity generally occurs when these regulatory mechanisms are overwhelmed by excessive exposure (68–70). Normal Cu balance is controlled primarily through intestinal absorption and biliary excretion, which makes clinically significant Cu toxicity relatively uncommon under normal exposure conditions (68).

When excessive exposure does occur, Cu toxicity is primarily associated with oxidative cellular injury and hepatic, gastrointestinal, renal, hematologic, and cardiovascular effects (69, 71–73). These toxicological effects provide biological context for the environmental findings but should not be interpreted as demonstrated health outcomes in Galápagos residents. Rather, the elevated Cu concentrations identified in some environmental matrices support continued assessment of Cu bioavailability, transfer into locally produced food, and potential exposure pathways within the archipelago.

### 4.2 Limitations

It is important to emphasize that the present study is among the few adopting a One Health approach to heavy metals in the Galápagos, integrating environmental data, wildlife, and an analysis of potential implications for human populations. Nevertheless, the findings should be interpreted in light of the limited evidence currently available. Only sixteen studies met the eligibility criteria, most of which were observational and cross-sectional in design. In addition, substantial methodological heterogeneity was observed regarding the environmental matrices, biological species, analytical techniques, and reported outcomes, limiting direct comparisons across studies and precluding quantitative synthesis. Consequently, the conclusions of this review should be interpreted primarily as a comprehensive mapping of the available evidence and identification of research gaps rather than as definitive estimates of environmental or human health risk.

Several additional limitations were identified, including the predominance of cross-sectional studies lacking temporal trend data, the underrepresentation of remote islands, and the absence of atmospheric and groundwater sampling.

Although most evidence suggests predominantly natural sources of heavy metals due to the archipelago's volcanic activity, anthropogenic contributions are also discussed, particularly indicating greater exposure in marine organisms located near ports. The interpretation of health risk remains limited without studies capable of clearly delineating contamination sources and assessing the magnitude of contamination in relation to proximity to volcanic or anthropogenic activity. Additional structural limitations include the lack of baseline human biomonitoring and clinical outcome data, limited assessment of drinking-water exposure, and insufficient consideration of genetic, epigenetic, and nutritional susceptibility factors.

The protocol for this scoping review was not prospectively registered. Although prospective registration is not a mandatory requirement under PRISMA-ScR, it can enhance methodological transparency and reduce the potential for selective reporting. Therefore, the absence of prospective protocol registration should be considered when interpreting the findings of this review.

### 4.3 Public health recommendations

The evidence synthesized in this review demonstrates that heavy metals in the Galápagos circulate through a continuous environment–fauna–human system. Within the environment, volcanic activity, hydrothermal systems, and phosphate fertilizers generate a contamination substrate in soils, sediments, and water (46–49). In fauna, metals progressively bioaccumulate from invertebrates to top predators, including fish (19, 34, 39, 40, 43), crustaceans (42), birds (44, 74), and pinnipeds (45), affecting animal health and ecosystem function. Moreover, these contaminants may modulate the immune systems of aquatic organisms, influencing susceptibility to infectious diseases with potential zoonotic implications (75).

At the human level, the population (approximately 28,583 inhabitants) is exposed at the terminus of multiple pathways through the consumption of contaminated fish and seafood, crops grown in metal-enriched soils, and the potential inhalation of volcanic particulates. The absence of human biomonitoring data, longitudinal studies, and chemical speciation analyses limits the accurate estimation of population-level risk. Under a One Health framework, it is therefore essential to integrate environmental surveillance with human epidemiological assessment and wildlife monitoring, in order to develop policies that simultaneously protect human health, biodiversity, and the sustainability of the insular ecosystem (51).

Based on the available evidence, the establishment of an interinstitutional surveillance and response framework is recommended, aimed at the identification, monitoring, and management of heavy metal exposure in the Galápagos Archipelago. This framework should integrate key stakeholders, including the Ministry of Public Health (MSP), the Galápagos National Park Directorate, and national and international research and higher education institutions, with the objective of coordinating actions in environmental monitoring, research, and public health protection under a One Health approach.

As a priority measure, the implementation of a population-based cross-sectional survey is recommended to characterize fish consumption patterns among residents, in order to identify the main dietary exposure pathways. This strategy should be complemented by a baseline human biomonitoring program, particularly targeting vulnerable groups such as children, women of reproductive age, and populations with high consumption of marine resources, with the aim of establishing reference exposure levels and strengthening epidemiological surveillance. Additionally, the installation of a pilot air quality monitoring station in Puerto Ayora is recommended to generate baseline data on atmospheric pollutants associated with both natural sources, such as volcanic activity, and anthropogenic sources.

In the medium and long term, it is essential to develop national guidelines for safe fish consumption tailored to the specific context of the Galápagos Archipelago, based on local scientific evidence regarding heavy metal concentrations in marine species and population consumption patterns. Likewise, strengthening local technical capacities through training programs in environmental health, biomonitoring, and contaminant surveillance is recommended to ensure the sustainability of monitoring and response activities (76, 77).

Studies specifically focused on water accessibility and water quality were outside the predefined eligibility criteria and were therefore not included in the evidence synthesis. Nevertheless, water quality represents an important knowledge gap within the One Health framework in the Galápagos Archipelago. This gap is particularly relevant because natural freshwater availability is limited across the archipelago, and several inhabited islands, including Santa Cruz and Isabela, rely substantially on brackish groundwater and alternative water supplies (78).

Existing water-quality studies have included metals or trace elements among the parameters assessed in urban, natural, and agricultural water sources; however, these investigations were not specifically designed to characterize heavy-metal contamination and provide limited information on individual metal concentrations (77, 79–81). Future research should therefore systematically assess heavy metals in drinking water, groundwater, and other relevant water sources to determine whether water represents an additional pathway of environmental and human exposure.

The integration of environmental monitoring and epidemiological surveillance into a unified One Health surveillance system is recommended. Such a system would enable continuous tracking of environmental contaminants, early identification of exposure risks, and the development of evidence-based public policies aimed at protecting human health, conserving ecosystems, and ensuring environmental sustainability in the Galápagos Archipelago.

## 5 Conclusions

This scoping review synthesizes the available evidence on the presence, distribution, and effects of heavy metals in the Galápagos Archipelago from a One Health perspective. The findings indicate that heavy metals are present across multiple environmental matrices and wildlife, with consistent evidence of bioaccumulation and biomagnification throughout trophic networks, particularly in marine species of dietary relevance.

Although several studies reported concentrations exceeding international guideline values in commercially important species, no primary studies evaluating human biomonitoring or direct health outcomes were identified. Therefore, the human health dimension of the One Health framework should currently be interpreted as a critical evidence gap, with potential risks inferred from environmental contamination and food-chain exposure rather than demonstrated through direct human data.

These findings highlight the need for integrated environmental, wildlife, and human health surveillance programs, as well as standardized long-term monitoring of heavy metals across environmental matrices. Future research should prioritize human biomonitoring, epidemiological studies, and multidisciplinary investigations to fully operationalize the One Health approach and strengthen evidence-based policies for environmental conservation and public health in the Galápagos Archipelago.

## Data Availability

The original contributions presented in the study are included in the article/Supplementary material, further inquiries can be directed to the corresponding author.
