# Supplementary material for: Heavy metals in the Galápagos Archipelago: a One Health scoping review
Source: Front Public Health. 2026 Oct 28;14:1896419. doi: 10.3389/fpubh.2026.1896419 (PMC13752062; doi:10.3389/fpubh.2026.1896419)
Supplement: Supplementary file 1 [file Data_Sheet_1.zip › Data Sheet Galapagos/Supplementary Table 1,2,3.DOCX]

Supplementary Material

# Table 1. Preferred Reporting Items for Systematic reviews and Meta-Analyses extension for Scoping Reviews (PRISMA-ScR) Checklist

| **SECTION** | **ITEM** | **PRISMA-ScR CHECKLIST ITEM** | **REPORTED ON PAGE #** |
| --- | --- | --- | --- |
| **TITLE** | | | |
| Title | 1 | Identify the report as a scoping review. | 1 |
| **ABSTRACT** | | | |
| Structured summary | 2 | Provide a structured summary that includes (as applicable): background, objectives, eligibility criteria, sources of evidence, charting methods, results, and conclusions that relate to the review questions and objectives. | 1-2 |
| **INTRODUCTION** | | | |
| Rationale | 3 | Describe the rationale for the review in the context of what is already known. Explain why the review questions/objectives lend themselves to a scoping review approach. | 2 |
| Objectives | 4 | Provide an explicit statement of the questions and objectives being addressed with reference to their key elements (e.g., population or participants, concepts, and context) or other relevant key elements used to conceptualize the review questions and/or objectives. | 3 |
| **METHODS** | | | |
| Protocol and registration | 5 | Indicate whether a review protocol exists; state if and where it can be accessed (e.g., a Web address); and if available, provide registration information, including the registration number. | N/A |
| Eligibility criteria | 6 | Specify characteristics of the sources of evidence used as eligibility criteria (e.g., years considered, language, and publication status), and provide a rationale. | 3 |
| Information sources* | 7 | Describe all information sources in the search (e.g., databases with dates of coverage and contact with authors to identify additional sources), as well as the date the most recent search was executed. | 3-4 |
| Search | 8 | Present the full electronic search strategy for at least 1 database, including any limits used, such that it could be repeated. | 4 |
| Selection of sources of evidence† | 9 | State the process for selecting sources of evidence (i.e., screening and eligibility) included in the scoping review. | 4 |
| Data charting process‡ | 10 | Describe the methods of charting data from the included sources of evidence (e.g., calibrated forms or forms that have been tested by the team before their use, and whether data charting was done independently or in duplicate) and any processes for obtaining and confirming data from investigators. | 4 |
| Data items | 11 | List and define all variables for which data were sought and any assumptions and simplifications made. | 4 |
| Critical appraisal of individual sources of evidence§ | 12 | If done, provide a rationale for conducting a critical appraisal of included sources of evidence; describe the methods used and how this information was used in any data synthesis (if appropriate). | 5 |
| Synthesis of results | 13 | Describe the methods of handling and summarizing the data that were charted. | 4 |
| **RESULTS** | | | |
| Selection of sources of evidence | 14 | Give numbers of sources of evidence screened, assessed for eligibility, and included in the review, with reasons for exclusions at each stage, ideally using a flow diagram. | 5 |
| Characteristics of sources of evidence | 15 | For each source of evidence, present characteristics for which data were charted and provide the citations. | 6 |
| Critical appraisal within sources of evidence | 16 | If done, present data on critical appraisal of included sources of evidence (see item 12). | 7-9 |
| Results of individual sources of evidence | 17 | For each included source of evidence, present the relevant data that were charted that relate to the review questions and objectives. | Table 2 |
| Synthesis of results | 18 | Summarize and/or present the charting results as they relate to the review questions and objectives. | 9-11 |
| **DISCUSSION** | | | |
| Summary of evidence | 19 | Summarize the main results (including an overview of concepts, themes, and types of evidence available), link to the review questions and objectives, and consider the relevance to key groups. | 18-21 |
| Limitations | 20 | Discuss the limitations of the scoping review process. | 21-22 |
| Conclusions | 21 | Provide a general interpretation of the results with respect to the review questions and objectives, as well as potential implications and/or next steps. | 23-24 |
| **FUNDING** | | | |
| Funding | 22 | Describe sources of funding for the included sources of evidence, as well as sources of funding for the scoping review. Describe the role of the funders of the scoping review. | 24 |

JBI = Joanna Briggs Institute; PRISMA-ScR = Preferred Reporting Items for Systematic reviews and Meta-Analyses extension for Scoping Reviews.

* Where *sources of evidence* (see second footnote) are compiled from, such as bibliographic databases, social media platforms, and Web sites.

† A more inclusive/heterogeneous term used to account for the different types of evidence or data sources (e.g., quantitative and/or qualitative research, expert opinion, and policy documents) that may be eligible in a scoping review as opposed to only studies. This is not to be confused with *information sources* (see first footnote).

‡ The frameworks by Arksey and O’Malley (6) and Levac and colleagues (7) and the JBI guidance (4, 5) refer to the process of data extraction in a scoping review as data charting*.*

§ The process of systematically examining research evidence to assess its validity, results, and relevance before using it to inform a decision. This term is used for items 12 and 19 instead of "risk of bias" (which is more applicable to systematic reviews of interventions) to include and acknowledge the various sources of evidence that may be used in a scoping review (e.g., quantitative and/or qualitative research, expert opinion, and policy document).

*From:* Tricco AC, Lillie E, Zarin W, O'Brien KK, Colquhoun H, Levac D, et al. PRISMA Extension for Scoping Reviews (PRISMAScR): Checklist and Explanation. Ann Intern Med. 2018;169:467–473. [doi: 10.7326/M18-0850](http://annals.org/aim/fullarticle/2700389/prisma-extension-scoping-reviews-prisma-scr-checklist-explanation).

# Table 2. Search Strategies used in the databases

| Database | Search Strategy |
| --- | --- |
| Pubmed | ("Cadmium"[Mesh] OR cadmium OR Cd OR "Mercury"[Mesh] OR mercury OR methylmercury OR Hg OR MeHg OR "Lead"[Mesh] OR lead OR Pb OR "Arsenic"[Mesh] OR arsenic OR As OR "Chromium"[Mesh] OR chromium OR Cr OR "Nickel"[Mesh] OR nickel OR Ni OR "Metals, Heavy"[Mesh] OR "heavy metal*" ) AND ( "Environmental Pollution"[Mesh] OR "Environmental Exposure"[Mesh] OR "Water Pollutants"[Mesh] OR "Soil Pollutants"[Mesh] OR pollution OR contamination OR "environmental contamination" OR exposure OR toxicity OR ecotoxicology OR bioaccumulation OR biomagnification OR bioconcentration OR "environmental monitoring" OR "risk assessment" OR "ecosystem*" OR "food chain" OR "trophic level*" OR "marine pollution" OR "aquatic environment*" OR "terrestrial ecosystem*" ) AND ( "Galapagos Islands"[Mesh] OR Galapagos OR Galápagos OR "Galapagos Islands" OR "Islas Galapagos" OR Ecuador ) |
| Scopus | TITLE-ABS-KEY(("cadmium" OR Cd OR "mercury" OR methylmercury OR Hg OR MeHg OR "lead" OR Pb OR "arsenic" OR As OR "chromium" OR Cr OR "nickel" OR Ni OR "heavy metal*") AND ("environmental pollution" OR "environmental exposure" OR "water pollutants" OR "soil pollutants" OR pollution OR contamination OR "environmental contamination" OR exposure OR toxicity OR ecotoxicology OR bioaccumulation OR biomagnification OR bioconcentration OR "environmental monitoring" OR "risk assessment" OR "ecosystem*" OR "food chain" OR "trophic level*" OR "marine pollution" OR "aquatic environment*" OR "terrestrial ecosystem*") AND ("Galapagos Islands" OR Galapagos OR Galápagos OR "Islas Galapagos" OR Ecuador)) |
| Web of Science | TS=(("cadmium" OR Cd OR "mercury" OR methylmercury OR Hg OR MeHg OR "lead" OR Pb OR "arsenic" OR As OR "chromium" OR Cr OR "nickel" OR Ni OR "heavy metal*") AND ("environmental pollution" OR "environmental exposure" OR "water pollutants" OR "soil pollutants" OR pollution OR contamination OR "environmental contamination" OR exposure OR toxicity OR ecotoxicology OR bioaccumulation OR biomagnification OR bioconcentration OR "environmental monitoring" OR "risk assessment" OR "ecosystem*" OR "food chain" OR "trophic level*" OR "marine pollution" OR "aquatic environment*" OR "terrestrial ecosystem*") AND ("Galapagos Islands" OR Galapagos OR Galápagos OR "Islas Galapagos" OR Ecuador)) |

**Table 3.** Characteristics of included studies

| **Author, Year** | **Article Title** | **Sampling Site** | **Study Design** | **Matrix / Population** | **Metal(s) Assessed** | **Analytical Method** | **Outcome** |
| --- | --- | --- | --- | --- | --- | --- | --- |
| Franco-Fuentes et al., 2021 [33] | Metals in commercial fish in the Galapagos Marine Reserve: Contribution to food security and toxic risk assessment | Galápagos Marine Reserve, Puerto Ayora (Santa Cruz), south-central-eastern region | Analytical observational cross-sectional | Muscle tissue of commercial fish (*Caulolatilus princeps*, *Mycteroperca olfax*, *Thunnus albacares*, *Seriolella violacea*) | Al, B, Ba, Cd, Ni, Pb, Sr, Ca, Cr, Cu, Fe, K, Li, Mg, Mn, Mo, Na, V, Zn | ICP-OES (Inductively Coupled Plasma Optical Emission Spectrometry) | Elevated Cd concentrations were detected in demersal species, with potential toxicological risk in *Mycteroperca olfax*; demersal species showed higher levels of Cr and Mo, whereas pelagic species exhibited higher Zn levels. The probable source is the volcanic origin of the archipelago. |
| Franco-Fuentes et al., 2021 [37] | Metal and metalloids concentration in Galapagos fish liver and gonad tissues | Galápagos Marine Reserve, Pelikan Bay, Puerto Ayora, Santa Cruz Island (south-central-eastern bioregion) | Analytical observational cross-sectional | Hepatic and gonadal tissues of demersal and pelagic fish (*Thunnus albacares*, *Seriolella violacea*, *Acanthocybium solandri*, *Caulolatilus princeps*, *C. affinis*, *Mycteroperca olfax*, *Pontinus clemensi*, *Hyporthodus mystacinus*, *Paralabrax albomaculatus*) | Al, B, Ba, Cd, Co, Ni, Pb, Sr, Ca, Cu, Cr, Fe, K, Li, Mn, Mo, Mg, Na, V, Zn | ICP-OES (Inductively Coupled Plasma Optical Emission Spectrometry) | High Cd concentrations were detected in the liver of demersal species (especially *Caulolatilus* spp. and *Paralabrax albomaculatus*), with potential physiological and reproductive risk; redistribution of Zn toward gonads associated with reproductive processes; higher metal concentrations in demersal species likely linked to the volcanic origin of the archipelago. |
| Franco-Fuentes et al., 2023 [38] | Analysis of metals and metalloids in commercial fish species from the Galapagos Marine Reserve: Toxicological and nutritional assessment | Galápagos Marine Reserve, Pelikan Bay, Puerto Ayora, Santa Cruz Island (south-central-eastern bioregion) | Analytical observational cross-sectional | Muscle tissue of pelagic and demersal commercial fish (*Acanthocybium solandri*, *Caulolatilus affinis*, *Pontinus clemensi*, *Hyporthodus mystacinus*, *Paralabrax albomaculatus*) | Al, B, Ba, Cd, Ni, Pb, Sr, Ca, Co, Cr, Cu, Fe, K, Li, Mg, Mn, Mo, Na, V, Zn | ICP-OES (Inductively Coupled Plasma Optical Emission Spectrometry) | All species exceeded the maximum permissible Cd limits according to EFSA standards; *Caulolatilus affinis* presented the highest toxicological risk and should not be commercialized. Demersal species exhibited higher metal concentrations than pelagic species, likely associated with the volcanic origin of the archipelago. Some species provide relevant nutritional value (Ca, Fe, Zn), but with toxicological risk associated with Cd. |
| Franco-Fuentes et al., 2023 [18] | Mercury in fish tissues from the Galapagos Marine Reserve: Toxic risk and health implications | Galápagos Marine Reserve (south-central-eastern and western bioregions); southern Isabela, northeastern San Cristóbal, and Pelikan Bay (Puerto Ayora, Santa Cruz) | Analytical observational cross-sectional | Muscle, hepatic, and gonadal tissues of 9 commercial pelagic and demersal species | Total Hg (assumed as methylmercury for risk assessment) | Acid digestion + CV-AAS with FIAS system (Cold Vapor Atomic Absorption Spectrophotometry) | Demersal species showed higher Hg concentrations than pelagic species. *Acanthocybium solandri*, *Pontinus clemensi*, and *Hyporthodus mystacinus* exceeded international regulatory limits (EFSA/FDA/Codex). *H. mystacinus* presented the highest toxicological risk (MoS > 1 up to 100 kg body weight). A volcanic origin is suggested as the primary source. Potential implications for human health and fish reproductive function were identified. |
| Garcia-Garin et al., 2024 [39] | Concentrations of lead in pinniped bones confirm Galapagos as a relatively unpolluted environment | Galápagos Archipelago (breeding sites on five islands); comparison with Mauritania and Uruguay | Analytical observational cross-sectional comparative | Bone (nasal turbinate) of Galápagos sea lions (*Zalophus wollebaeki*, n=20) | Pb | ICP-MS (Inductively Coupled Plasma Mass Spectrometry) | Pb concentrations in the Galápagos were significantly lower than in Mauritania and Uruguay, confirming the archipelago as a relatively pristine environment. Pinnipeds proved to be effective bioindicators of marine contamination. Detected levels did not exceed toxicological thresholds, suggesting low local anthropogenic impact. |
| Zarn et al., 2020 [40] | Stable isotope and mercury analyses of the Galapagos Islands seabird community | Galápagos Archipelago: Genovesa, North Seymour, Daphne Major, South Plaza, San Cristóbal (Punta Pitt), Floreana, Española (Punta Suárez and Punta Cevallos) | Analytical observational longitudinal | Pectoral feathers of adult seabirds (8 species: *Sula sula*, *S. granti*, *S. nebouxii*, *Fregata minor*, *F. magnificens*, *Oceanodroma tethys*, *Creagrus furcatus*, *Phaethon aethereus*) | Total Hg | Direct mercury analyzer DMA-80 (Direct Mercury Analyzer) | Elevated Hg concentrations were observed in several species, with higher levels during La Niña events associated with increased ocean upwelling. Hg showed bioaccumulation at higher trophic levels and variation according to location and diet. Some colonies exhibited greater exposure, indicating potential ecological risk and supporting the use of seabirds as bioindicators. |
| Jiménez et al., 2020 [41] | Trace metals (Hg, Pb, and Cd) in feathers of four Galapagos waterbird species | Galápagos Archipelago: Isabela (Caleta Iguana, Puerto Pajas, Marielas Islets), Fernandina (Playa Escondida, Carlos Valle), Española (Punta Suárez), Santa Cruz, Floreana, Santiago, Rábida, Bainbridge | Analytical observational cross-sectional | Feathers of waterbirds (*Spheniscus mendiculus*, *Phalacrocorax harrisi*, *Phoebastria irrorata*, *Phoenicopterus ruber*) | Hg, Pb, Cd | Potentiometric electrochemical analysis for Hg (Potentiostat µStat400) and flame atomic absorption spectrophotometry (FAAS) for Pb and Cd | Hg was not detectable in any species. Pb and Cd were primarily detected in flamingos, with lower levels compared to other global ecosystems. The observed contamination likely has a natural volcanic origin rather than an anthropogenic source. Birds proved to be effective bioindicators of environmental contamination. |
| Dinter et al., 2021 [42] | Heavy metal contents, mobility and origin in agricultural topsoils of the Galapagos Islands | Agricultural zones of Isabela, Santa Cruz, and San Cristóbal (0–20 cm depth) | Analytical observational cross-sectional (geochemical) | Agricultural topsoil; n = 125 composite samples | Cd, Co, Cr, Cu, Ni, Pb, U, Zn | Aqua regia acid digestion + ICP-MS (metals) and ICP-OES (P); NH₄NO₃ extraction for mobile fraction | High concentrations of Cd, Co, Cr, Cu, Ni, and Zn were observed, many exceeding international threshold values, indicating potential ecological risk. Ni, Co, and Cr increased with geological soil aging. Cd, Zn, and Cu showed anthropogenic influence, particularly from phosphate fertilizers. Cd exhibited increasing mobility and potential transfer to crops, with implications for animal and human health. |
| Alava-Jurado et al., 2025 [43] | Spatial variation in otolith elemental chemistry of *Stegastes arcifrons* reflects environmental patchiness at the Galápagos archipelago | Galápagos Marine Reserve: 14 sites including Fernandina, Floreana, San Cristóbal, Española, Pinzón, Baltra, Santiago, and Pinta | Analytical observational cross-sectional with environmental geochemical component | Otoliths (sagittae) of adult reef fish *Stegastes arcifrons* (n ≈ 10 per site) | Ba, Mg, Mn, Sr (expressed as Ba/Ca, Mg/Ca, Mn/Ca, Sr/Ca) | Acid digestion with HNO₃ + ICP-MS (Inductively Coupled Plasma Mass Spectrometry) | Significant differences in elemental ratios were observed among sites, reflecting environmental and oceanographic heterogeneity. Sr/Ca and Mn/Ca showed a positive correlation with sea surface temperature. Fish acted as bioindicators of environmental conditions and oceanographic variability. Otolith chemistry enabled the identification of upwelling gradients and water masses. |
| Savage et al., 2024 [44] | Assessing the chemical landscape of the Galápagos Marine Reserve | Galápagos Marine Reserve (GMR), including multiple islands and marine ecosystems | Systematic / narrative environmental review | Multiple environmental and biological matrices: water, sediments, soils, marine fauna (birds, fish, pinnipeds, reptiles) | Hg, Pb, Cd, Cr, Zn, and other heavy metals | Not applicable (review of previous studies using ICP-MS, AAS, DMA, among others) | The review identified a widespread but heterogeneous presence of heavy metals in the Galápagos ecosystem, with documented bioaccumulation in fish, birds, and marine mammals. Main sources include natural volcanic activity, agricultural runoff, ocean transport, and human activities. Ports and coastal areas were identified as contamination hotspots, with ecological implications and potential risks to human and environmental health. |
| Vega-Mariño et al., 2024 [45] | Review of One Health in the Galápagos Islands (Part 2): climate change, anthropogenic activities, and socioeconomic sustainability | Galápagos Archipelago (marine and terrestrial ecosystems) | Narrative review with a One Health approach | Multiple matrices: fish, birds, agricultural soils, water, and marine fauna | Hg, Pb, Cd, Cr, Cu, Ni, Zn, Co | Not applicable (synthesis of studies using ICP-MS, AAS, DMA, among others) | Bioaccumulation of heavy metals was documented in fish and birds, with biomagnification at higher trophic levels. Main sources include volcanic activity, upwelling currents, and the use of agrochemicals. Metal accumulation represents a risk to animal, environmental, and potentially human health under a One Health framework. |
| Muñoz-Abril et al., 2022 [46] | Elevated Mercury Concentrations and Isotope Signatures (N, C, Hg) in Yellowfin Tuna (*Thunnus albacares*) from the Galápagos Marine Reserve and Waters off Ecuador | Galápagos Marine Reserve (San Cristóbal and Santa Cruz Islands) and Ecuadorian mainland coast (Santa Rosa, Santa Elena) | Analytical observational cross-sectional (ecological) | Red muscle tissue from 347 yellowfin tuna (*Thunnus albacares*) collected during the cold (2015) and warm (2016) seasons | Total Hg | Direct Mercury Analyzer (DMA-80) for THg; stable isotope analyses (δ¹³C, δ¹⁵N, δ²⁰²Hg, Δ¹⁹⁹Hg, Δ²⁰⁰Hg) using isotope ratio mass spectrometry (IRMS) and MC-ICP-MS | THg concentrations ranged from 0.20–9.60 mg/kg wet weight, with no significant differences between the Galápagos Marine Reserve and the Ecuadorian mainland coast or between seasons. Mean THg was 1.72 ± 1.11 mg/kg in the Galápagos and 1.11 ± 1.67 mg/kg on the mainland coast. Stable isotope analyses indicated that mercury exposure is mainly associated with marine food-web dynamics, atmospheric deposition, and regional upwelling processes, while isotopic differences reflected variation in photochemical mercury processing rather than different contamination sources. |
| Rechberger et al., 2021 [47] | Cadmium retention and microbial response in volcanic soils along gradients of soil development in the Galápagos Islands | Santa Cruz Island, Galápagos (altitudinal gradients and chronosequences of volcanic soils) | Experimental geochemical laboratory study | Volcanic agricultural and natural soils at different stages of pedogenic development | Cd | Acid digestion + ICP-MS; adsorption and desorption experiments; molecular microbiological analysis | Volcanic soils exhibited a high capacity for Cd retention, particularly in young soils rich in allophanes. Retention decreased with soil age, increasing Cd mobility and potential bioavailability. Cd exposure significantly affected microbial activity, with implications for soil fertility and the risk of transfer to crops and trophic chains. |
| Landolfi et al., 2025 [48] | Life on the edge: mineral incrustations colonized by fungal communities in the sulfur fumarole on Sierra Negra volcano (Galápagos Archipelago) | Sierra Negra Volcano, Isabela Island, Galápagos (active fumarole with gypsum mineral incrustations) | Analytical observational microbiological and geochemical | Gypsum mineral incrustations colonized by fungal communities | Al, Fe, Zn, Mn, Cu, Mg, Na, K, P | X-ray diffraction (XRD) for mineralogy; elemental analysis via chemical digestion and elemental spectrometry | Variable concentrations of trace metals (Al, Fe, Zn, Mn, Cu) were detected in volcanic incrustations, significantly influencing the composition of fungal communities. Metals and nutrients acted as key determinants of microbial structure. Volcanic fumaroles represent active geochemical reservoirs with implications for biogeochemical cycles and the environmental mobilization of metals. |
| Jackson et al., 2024 [49] | Mercury Levels in the Crab *Grapsus grapsus* across the Galápagos Archipelago | Galápagos: Academy Bay (Santa Cruz, urban), South Plaza (protected), San Cristóbal (urban, near the dock), Isabela (urban, near the dock), Isabela (protected: Playa del Amor) | Analytical observational cross-sectional | Leg muscle tissue of crab *Grapsus grapsus* (n = 52 total; per site n = 10 except Academy Bay n = 12). Also: barnacles *Tetraclita milleporosa* (n = 20) and oysters *Saccostrea palmula* (n = 10) | Total Hg | Direct Hg analyzer Milestone DMA-80 evo (Direct Mercury Analyzer) | Hg concentrations varied significantly by site, with the highest levels in San Cristóbal (mean 2.04 mg/kg dw; 0.51 mg/kg ww) and South Plaza (1.19 mg/kg dw; 0.30 mg/kg ww). When converted to wet weight, San Cristóbal and South Plaza were within or above ranges (0.3–0.5 mg/kg ww) considered potentially harmful; all crabs from San Cristóbal and 50% from South Plaza exceeded the EPA threshold (0.3 mg/kg ww). Possible sources include volcanism and/or local anthropogenic inputs. |
| Goff et al., 2000 [50] | Contrasting hydrothermal activity at Sierra Negra and Alcedo volcanoes, Galápagos Archipelago, Ecuador | Sierra Negra and Alcedo volcanoes, Isabela Island, Galápagos | Analytical observational geochemical | Fumarolic gases, hydrothermal condensates, altered rocks, and mineral deposits | Fe, Zn, Cu, and other trace metals associated with hydrothermal deposits | Elemental and isotopic geochemical analysis of gases and condensates (including elemental spectrometry and stable isotope analysis) | Trace metal anomalies were detected in hydrothermal deposits and volcanic alteration products, indicating active circulation of hydrothermal fluids capable of mobilizing metals. Hydrothermal systems are controlled by structural faults and contribute to the natural redistribution of metals in the volcanic environment of the Galápagos. |

**Abbreviations**: ICP-MS, inductively coupled plasma mass spectrometry; ICP-OES, inductively coupled plasma optical emission spectrometry; AAS, atomic absorption spectrometry; CV-AAS, cold vapor atomic absorption spectrometry; DMA, direct mercury analyzer; Hg, mercury; Cd, cadmium; Pb, lead; Zn, zinc; Cu, copper; Cr, chromium; Ni, nickel; Fe, iron; Al, aluminum; Mn, manganese; Sr, strontium. * Studies and specific metals were prioritized based on their reporting frequency and their potential to exceed regulatory safety thresholds.
